# Persistent transmission of soil-transmitted helminths despite 16 years of uninterrupted Mebendazole- and ivermectin-based preventive chemotherapy in the Lomie Health District (East Region, Cameroon): The emergency of complementary control strategies

**DOI:** 10.1371/journal.pntd.0012508

**Published:** 2024-09-25

**Authors:** Arnauld Efon-Ekangouo, Virtue F. De-Gaulle, Yannick Emalio, Verner N. Orish, Linda Djune-Yemeli, Michèle L. Simo-Simo, Martine A. Tsasse, Laetitia Mbakam, Hugues C. Nana-Djeunga, Joseph Kamgno

**Affiliations:** 1 Department of Biochemistry, Faculty of Sciences, University of Yaoundé I, Yaoundé, Cameroon; 2 Higher Institute of Scientific and Medical Research (ISM), Yaoundé, Cameroon; 3 Department of Public Health, Faculty of Medicine and Biomedical Sciences, University of Yaoundé I, Yaoundé, Cameroon; 4 Unversity of Health and Allied Sciences, Ho Volta Region, Ghana; 5 Research Centre for Epidemiology, Biostatistics and Clinical Research, Université Libre de Bruxelles, Brussels, Belgium; 6 Department of Microbiology and Parasitology, Faculty of Science, University of Buea, Buea, Cameroon; Consejo Nacional de Investigaciones Cientificas y Tecnicas, Fundación Mundo Sano, ARGENTINA

## Abstract

**Background:**

The control of the Soil-Transmitted Helminths (STH) infections primarily relies on the school-based Preventive Chemotherapy (PCT) with mebendazole. Given the efficacy of ivermectin on STH, the control of the latter is expected to be potentialized in areas where ivermectin is also distributed for onchocerciasis and/or lymphatic filariasis control/elimination. This study aimed to assess the prevalence and intensity of STH in the Lomie Health District where annual school-based deworming campaigns and community-directed treatments with Ivermectin have been underway for almost two decades.

**Methodology/principal findings:**

A quantitative cross-sectional study was conducted in 10 schools of the Lomie Health District, East Region, Cameroon. Stool samples were collected from school-aged children and analysed using the Kato-Katz technique. Semi-structured questionnaires were administered to enrolees to assess compliance with water, sanitation, and hygiene (WASH). Of the 491 children (median age: 9 years; IQR: 7–10) enrolled, 83.9% (95% CI: 80.3–87.1) were infected with at least one STH species. *Trichuris trichiura* was the predominant species (78.5%), and no hookworm was found. The prevalence trend slightly decreased between 1987 and 2010 (~8%) and remained unchanged since 2010 (*p-value* = 0.05). Overall, 46.8% and 41.8% of children were heavy-to-moderately infected with *Ascaris lumbricoides* and *T*. *trichiura*. Poor hand hygiene (OR: 2.24, 95% IC: 1.4–3.4, *p-value* = 0.0002) and the use of river as a source of drinking water (OR: 14.8, 95% IC: 6.9–33.3, *p-value* = 0.0001) were the main risk factors associated with the STH infection in Lomie Health District.

**Conclusions/significance:**

The persistent high prevalence and intensity of STH infection despite 16 years of mebendazole-based PCT and expected collateral impact of ivermectin mass distribution, points to plausible implementation gaps, poor compliance to WASH or sub-optimal efficacy of the anthelminthics used. This study highlights the need to further assess the cause of the persistent high prevalence and implement context-adapted control measures in order to curb STH transmission.

## Author summary

Soil-transmitted helminthiasis (STH) is a disease caused by intestinal worms, mainly common in impoverished settings lacking access to water, sanitation, and hygiene structures. Annual mass distribution of mebendazole to at risk groups is the main control strategy. Ivermectin, another community mass-distributed treatment for onchocerciasis and/or lymphatic filariasis control/elimination, also showed a collateral impact on STH control. The Lomie Health District is a predominantly rural setting where annual school-based deworming campaigns and/or community-based preventive chemotherapy with ivermectin for onchocerciasis are ongoing. This study aimed to assess the current prevalence and intensity of STH infection among school-aged children in the Lomie Health District. STH transmission remains substantially high despite the mebendazole-based annual deworming exercises in schools and community-based ivermectin preventive chemotherapy. The current prevalence of the infection is similar to the baseline level 16 years earlier, at the commencement of the MDA strategies. Further studies are needed to understand the contextual determinants of the high STH prevalence to inform targeted interventions to reducing the transmission of the infection in the Lomie Health District.

## Introduction

Soil-transmitted helminths (STHs) are a group of parasitic nematodes of significant public health importance in sub-Saharan Africa (SSA) [1]. Approximately, 1.5 billion people are estimated to be infected with at least one STH species worldwide [2]. The main species causing disease among humans are roundworms (*Ascaris lumbricoides* and *Strongyloides stercoralis*), whipworms (*Trichuris trichiura*) and hookworms (*Necator americanus* and *Ancylostoma duodenale*) [3,4]. These helminths cause significant morbidity, mainly in the younger population (pre and school-age children), with anaemia, malnutrition, impairment of cognitive development, and physical growth being some of the adverse effects [5]. Female worms produce eggs which are excreted in the faeces of infected people. Therefore, poor sanitation practices such as open defecation, coupled with a lack of appropriate water, sanitation, and hygiene (WASH) structures, facilitate transmission, which occurs either through oral ingestion of eggs or transdermal penetration of larvae [3]. Structural improvements in impoverished communities through the provision of adequate potable water and sanitary facilities are sustainable and long-term control measures [6,7]. However, this is usually unavailable in low-resourced settings, making STH a condition predominant in impoverished communities. The main control strategy recommended by the World Health Organization (WHO) emphasises morbidity control through mass-drug administration (MDA) of albendazole / mebendazole (ALB/MBZ-based MDA), targeting at-risk persons including pre-school and school-aged children living in endemic areas [8]. Since scaling up neglected tropical disease (NTD) control programmes across SSA using anti-helminthics in the year 2000, there has been a substantial decrease in the geographic extent and burden of STH from an estimated population-weighted prevalence of 44% in children aged 5–14 years in 2000 to 13% by 2018 [9]. However, STH infection is still a public health concern in most SSA countries.

In Cameroon, STH is country-wide distributed with an estimated number of 10 million people infected [10]. The national epidemiological survey conducted in 1987 prior to the implementation of control interventions showed a geographic discrepancy in the distribution of the disease with prevalence rates ranging from 2–99% [11]. The country has adopted the WHO recommended morbidity control strategy of ALB/MBZ-based MDA targeting at-risk persons [1,12] in all the endemic health districts throughout the country since 2007. The drugs used by the programme are those offered free of charge by *Johnson & Johnson*. The pre-school aged children and women are reached with the albendazole during the enlarged immunisation programme while school-aged children (5 to 14 years) are reached with the mebendazole treatment through school-based MDA, usually undertaken on an annual basis [10]. The nationwide average reported therapeutic coverage is estimated at 58.2%. Since the intensification of MBZ-based MDA through the deworming campaigns targeting at-risk groups in 2007, a substantial decrease has been noted in the geographical extent and burden of STH infections compared with the pre-intervention situation [13]. However, post-intervention data remain limited in areas of high endemicity, such as the Lomie Health District, East Region of the country. Noteworthy, the reduction of the STH prevalence appears to be greater in areas where deworming campaigns overlaps with community-directed interventions with ivermectin (IVM), a broad-spectrum anthelmintic drug used for the control/elimination of onchocerciasis and lymphatic filariasis [14–17]. Indeed, IVM has proved effective against STH, with an estimated reduction in prevalence of 84.5%, 49.9% and 35.3% for *S*. *stercoralis*, *T*. *trichiura* and *A*. *lumbricoides* respectively [18,19], and the community-based administration strategy enables to extend its impact to broader population [16]. Hence, in areas where the implementation of these two interventions overlaps, a potentiated effect described as “beneficial impact” of ivermectin on STH prevalence has been demonstrated [14,15,20–22]. This study sought to assess the level of transmission (prevalence and intensity) and the at-risk factors of STH infection amongst school-aged children in Lomie Health District (East Region, Cameroon) after 16 years of school-based mebendazole and community-based ivermectin mass administrations.

## Methods

### Ethics statement

The protocol of this study was approved by the Ethics Review and Consultancy Committee (ERCC)/Cameroon Bioethics Initiative (CAMBIN) (N° Ref CBI/494/ERCC/CAMBIN). The study was conducted in accordance with the Declaration of Helsinki. Participants were informed about the objectives of the study, the sampling procedures, the potential risks, the benefits and their right to freely withdraw their consent at any time. Formal written consent was obtained from the parent/guardian of children. A unique identifier (barcode) was attributed to each enrolee for confidentiality purpose, and all data generated were treated anonymously.

### Study area and history of treatments

**Fig 1:**
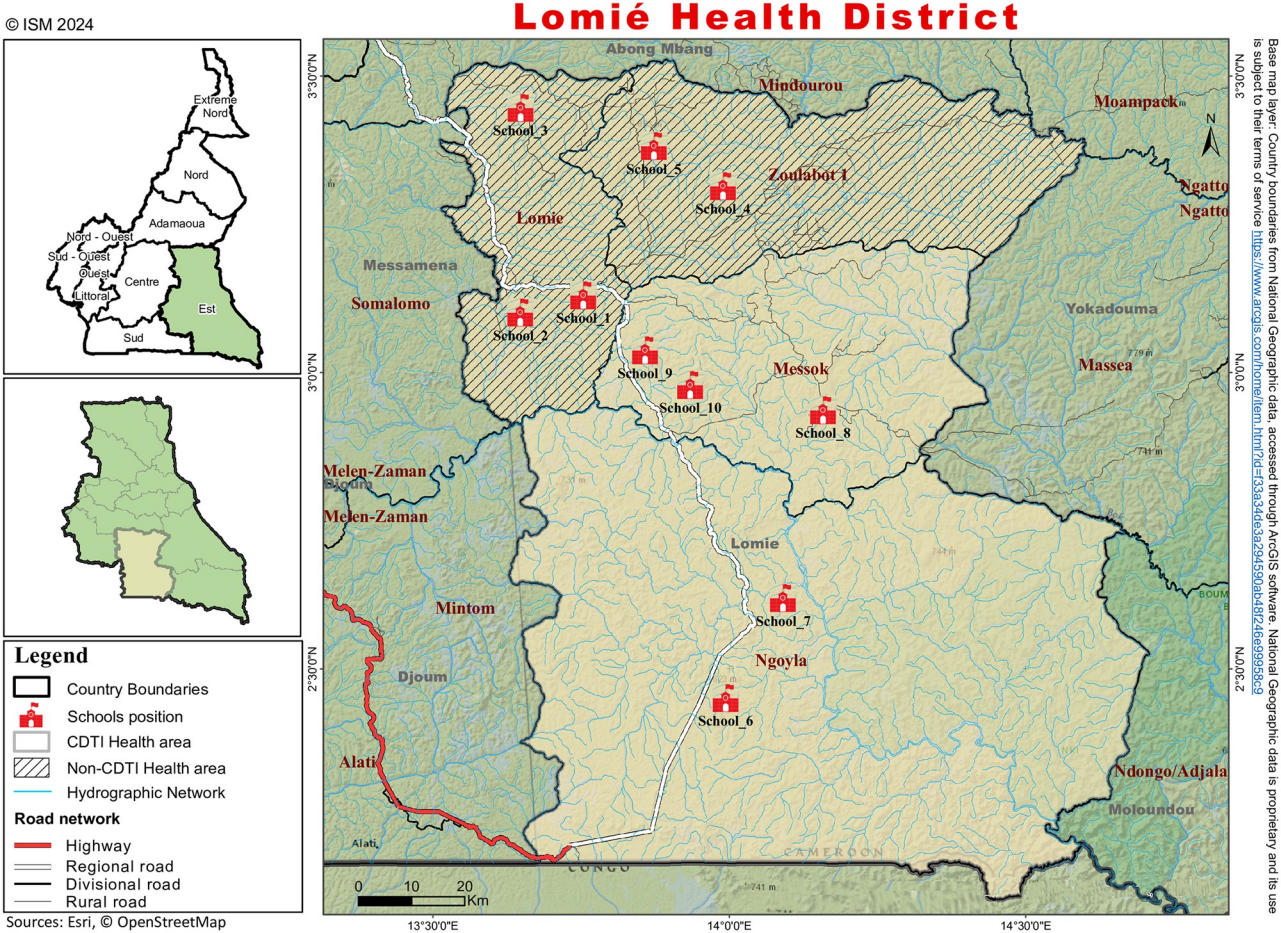
Lomie district map.

**Fig 2:**
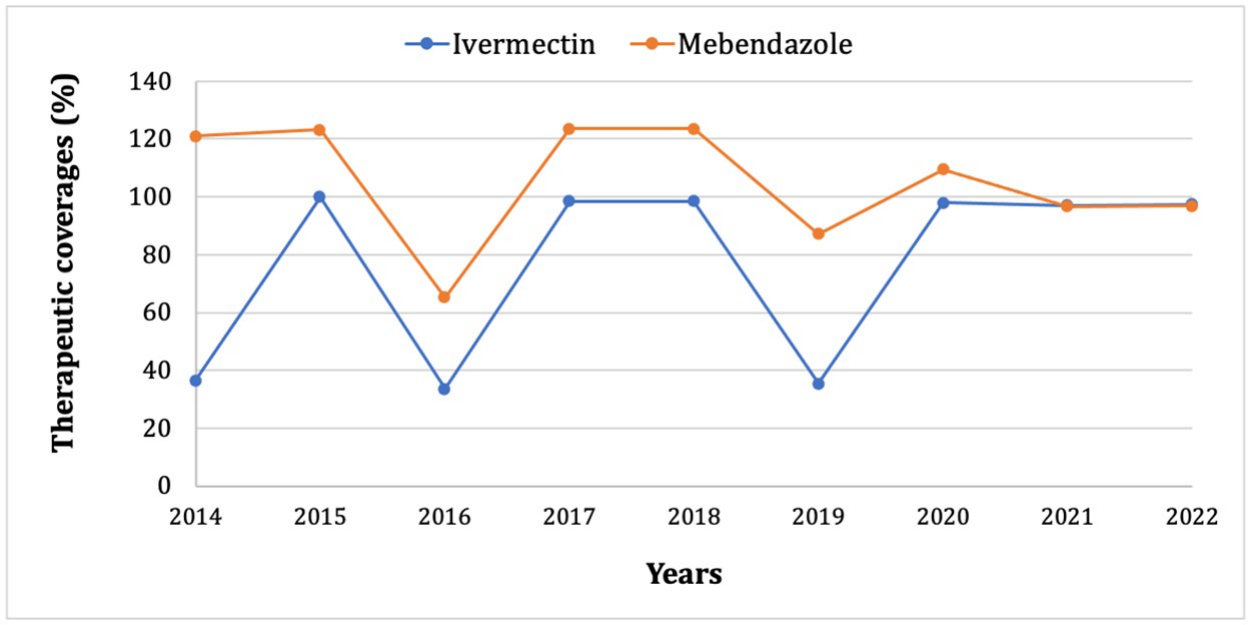
Trends in therapeutic coverage of mebendazole in school-age children and ivermectin in the eligible population of Lomie district over the nine years preceding the survey. A rate higher than 100% is due to the inaccuracy of the denominator used to calculate the coverage rate, as the number of children attending school is not updated regularly.

The study was carried out in the Lomie Health District, located in the Haut-Nyong Division, East Region, Cameroon, which belongs to a forest bioecological zone. The Lomie Health District is endemic for onchocerciasis and STH, two preventive chemotherapy-based neglected tropical diseases. The mass-treatment landscape for the control of these preventable NTDs in the health district is narrowly related to their geographic distribution and the level of endemicity. On the basis of the pre-interventional data (baseline), the whole district has been treated for the STH with mebendazole via a school-based MDA targeting school-aged children during the annual deworming campaigns. In addition, the pre-school age children and childbearing women are treated with albendazole during the extended immunisation program. While mebendazole/albendazole is administered in the whole district (100% of geographic coverage), the community-directed treatment with ivermectin (CDTI) against onchocerciasis is implemented according to the epidemiological landscape of onchocerciasis and loiasis. In order to mitigate the risk of the severe adverse events (SAEs) associated with the ivermectin treatment in people heavily infected with loiasis (another filariasis endemic in the health district) as described by Chippaux *et al*. [23–25], the Lomie health district is only partially treated with ivermectin, i.e. only areas that are highly or moderately endemic to onchocerciasis are treated and areas that are low-endemic are not treated [26]. Therefore, the district’s health map (Fig 1), which comprises four health areas, is structured into two clusters; Lomie and Zoulabot 1 in the North of the district, which are hypo-endemic for onchocerciasis, hence excluded from mass treatment with ivermectin (non-CDTI health areas) and the Messok and Ngoyla health areas in the Centre and South of the district (close to the *Dja* River) which are meso-endemic for onchocerciasis and are under CDTI (CDTI health areas). The CDTI and school-based MDA with mebendazole have been ongoing respectively for 23 and 16 years in Lomie District. The assessment of the trend in therapeutic coverage of the two drugs in the district over the nine years preceding the study using the ESPEN (Expanded Special Project for Elimination Neglected Tropical Diseases) database (https://espen.afro.who.int/), showed a variability throughout the years in therapeutic coverages with estimated average coverages of 105.1% for mebendazole in school-age children and 77.2% for ivermectin in the eligible populations (Fig 2).

### Study design and sample size

This was a cross-sectional study carried out from April to June 2023. The Lomie Health District was purposively chosen based on its endemicity to STH and onchocerciasis, and therefore undergoing annual MDA using mebendazole and ivermectin. The sample size of the study was estimated using the formula described for estimating the proportion of binary outcome-descriptive study [27,28]. $N=\frac{Z^{2}*P(1-P)}{D^{2}}$, where N = minimum number of sample size, Z^2^ = standard value, P = expected prevalence of intestinal parasitic infections in the study area and D = marginal error, at 95% confidence interval: Z = 1.96 and D = 0.05 since no report was recorded for infection prevalence of intestinal parasitic infections in the area, P = 50%. Accordingly, N = (1.96)^2^ x 0.5 (0.5)/ (0.05)^2^ = 384. The sampling was conducted in primary schools in all the health areas to establish a fine mapping of the STH at the Health District level. Briefly, the health areas of the district were purposively organised into two clusters based on CDTI implementation status, the non-CDTI health areas (Lomie and Zoulabot 1 health areas) the CDTI health areas (Messock and Ngoyla health areas). In each cluster, 05 schools were randomly selected from the overall list of 21 primary schools that account the district (13 and 08 schools in CDTI zone and in non-CDTI zone) (Fig 1). In each cluster, 05 schools were randomly selected from the overall list of 21 primary schools in the district (Fig 1). Overall, ten (10) primary schools were selected and around 50 pupils (25 boys and 25 girls) enrolled in each school. The targeted population consisted of school-aged children (from 5 to 16 years old) based on their status of “at-risk groups for STH infection”, and being the target group for deworming campaigns.

### Data and sample collection and processing

In the schools, meetings were held with the headmasters/mistresses and teachers to explain the purpose of the survey, the impact of the diseases investigated in children’s health and education. Their assistance was also sought in organising the pupils for the data collection activities. A questionnaire designed to collect socio-demographic data, history of albendazole/mebendazole and ivermectin uptake as well as some risk factors that expose the STH infection was administered (S1 Text). The stool sample collection procedure was explained to each student after which a labelled 60 mL plastic screw-cap vial was given to selected pupils to be returned the next day with stool produced the morning of the sample collection. Stool sampled provided by students were checked for quantity and identification, and transported to the Lomie District Hospital in a cold box for immediate (same day) parasitological examination using the Kato-Katz technique as described by Montresor *et al* [29]. Briefly, the stool was homogenized and passed through a 50 μm pores to reduce big debris. The stool was calibrated (41.4 mg), deposed on a slide and recovered with cellophane paper impregnated with malachite green solution. The preparation was analysed under a microscope by experienced laboratory technicians, and results were reported as the number of eggs per gram of stool.

### Data management and statistical analysis

Field and laboratory data were collected electronically with digital tablets using the ODK collect application and was real-time uploaded onto the Linode online server and subsequently downloaded in csv file and imported into R software version (version 4.3.2) for statistical analyses. Categorial variable including presence of eggs in stool, at-risk behaviours, treatment history and CDTI status of the health area were expressed as the proportion/prevalence with the 95% confidence interval (CI) calculated using the exact method. Continuous variables including the age of participants, intensities of the STH infection were expressed as mean with standard deviation (SD) or the median with interquartile range (IQR) when the distribution was not normal. The intensity of STH infection was estimated as the arithmetic mean of the number of eggs per gram of faeces (epg) among school-aged children. The intensity of the STH infection was organised in classes defined for each parasite as “light”, “moderate” or “heavy” based on faecal egg counts using the cut-off threshold set by WHO [29]. Proportions/prevalence were compared between co-variates using Chi-square test (with Yates correction for continuity and Fisher exact test for small sample sizes). Intensities of infection were compared among age and sex classes using the non-parametric Mann-Whitney or Kruskal-Wallis tests, respectively. The Multivariate logistic regression model was used to explore the extent to which risk behaviours were associated with the prevalence and intensity of STH infection in the study population. The threshold for significance was set at 5% for all the statistical analyses.

## Results

### Study population characteristics and hygiene conditions

Overall, 491 school-aged children between the ages of 5 and 16 years (median: 9; IQR: 7–10) were enrolled in 10 schools (05 schools in CDTI health areas and 05 in non-CDTI health areas), a single private school (school 1, the only one found in the district) and 9 public schools. Female represented 47.4% of the overall population and gender distributions among the schools and CDTI vs non-CDTI areas were comparable (Chi-squared = 0.42, df = 1, *p-value = 0*.*51*). The Table 1 summarises the frequencies of assessed risk factors associated with the STH transmission at the school level of Lomie health district. Of the 10 schools surveyed, 02 schools (20%) had no latrines available for students and only 02 schools (belonging to the non-CDTI health areas) had functional handwashing facilities close to latrines. In the schools where latrines exist, the ratio of students to a toilet facility ranged from 1: 61 (1 toilet for 61 students) in School 4, to 1: 216 in school 2. In schools where the toilet facilities were absent, 88.2% of children declared their main alternative was to defecate in the bushes surrounding the schools. The hand hygiene practices were highly variable amongst schools. The appreciation of the hand cleanliness showed that 57.5% of children had dirty fingers. This proportion varied from 33.3% to 98.0% of children dirty handed (Chi-square = 97.9, df = 9 *p-value* < 0.0001). Fingernail biting was very common amongst the students, with 52.5% of the students stating they bite their nails, with a significant difference in proportion amongst schools (Chi-squared = 57.9, df = 9 *p-value* < 0.0001). Overall, 15.5% of children responded they never clean fruits and vegetables prior to eating. The comparison of the hygiene conditions according to the CDTI status showed that the proportion of children with dirty hands was significantly higher in non-CDTI areas (Chi- squared = 58.4, df = 1, *-value* < 0.0001), while no difference was observed in the fingernails biting behaviour in accordance to the CDTI status (Chi-squared = 1.8, df = 1, *p-value* = 0.18). The proportion of children who reported always walking barefoot, never wash their hands after using the toilet and lacking toilet facility at home, were significantly higher in CDTI areas (19.8%, 20.7% and 4.3% respectively) compared to non-CDTI areas (6.2%, 7.7 and 2.7% respectively) (Chi-squared = 32.634, df = 2, *p-value* < 0.0001).

**Table 1:** Availability of toilet facilities and hygiene practices amongst children.

Characteristics	CDTI status	Non–CDTI health areas						CDTI health areas						Total
	Health areas	Lomie		Zoulabot 1				Sub-Total	Ngoyla		Messock		Sub-Total	
	Schools	School 1	School 2	School 3	School 4	School 5	School 6		School 7	School 8	School 9	School 10		
Ratio (toilet available/children)		1/72	1/216	1/125	1/61	0	1/119	0/154	1/98	0/76	1/68	0	1/198	1/158
Handwashing facility		Yes	No	No	Yes	No	-	No	No	No	No	No	-	-
Toilet facility at home n (%)	No	1(2.0)	1(1.7)	2(4.2)	0(0.0)	3(5.6)	7(2.7)	1(2.1)	0(0.0)	4(7.8)	2(4.1)	3(8.8)	10(4.3)	17(3.5)
	Yes	49(98.0)	58(98.3)	47(95.8)	48(100)	51(94.4)	253(97.7)	46(97.9)	51(100.0)	47(92.2)	47(95.9)	31(91.2)	222(95.7)	474(96.5)
Alternative when toilet is absent at home n (%)	Nearby bush	1(100)	1(100)	1(50.0)	-	3(100)	6(85.7)	1(100)	-	4(100.0)	1(50.0)	3(100)	9(90.0)	15(88.2)
	Neighbours	0.0	0.0	0.0	-	0(0.0)	0(0.0)	0(0.0)	-	0(0.0)	1(50.0)	0(0.0)	1(10.0)	1(5.9)
	Other	0.0	0.0	1(50.0)	-	0(0.0)	1(14.3)	0(0.0)	-	0(0.0)	0(0.0)	0(0.0)	0(0.0)	1(5.9)
Fingernails cleanliness n (%)	Clean	33(66.0)	39(66.1)	32(66.7)	31(64.6)	17(31.5)	152(58.7)	10(21.3)	20(39.2)	1(2.0)	17(34.7)	8(23.5)	56(24.1)	208(42.4)
	Dirty	17(34.0)	20(33.9)	16(33.3)	17(35.4)	37(68.5)	107(41.3)	37(78.7)	31(60.8)	50(98.0)	32(65.3	26(76.5)	176(75.9)	283(57.6)
Fingernails bites n (%)	No	33(66.0)	22(37.3)	16(33.3)	22(45.8)	22(40.7)	115(44.4)	26(55.3)	28(54.9)	24(47.1)	38(77.6)	2(5.9)	118(50.9)	233(47.5)
	Yes	17(34.0)	37(62.7)	32(66.7)	26(54.2)	32(59.3)	144(55.6)	21(44.7)	23(45.1)	27(52.9)	11(22.4)	32(94.1)	32(49.1)	258(52.5)
Walk barefoot n (%)	Always	1(2.0)	3(5.1)	1(2.1)	5(10.4)	6(11.1)	16(6.2)	20(42.6)	0(0.0)	25(49.0)	0(0.0)	1(2.9)	46(19.8)	62(12.6)
	Never	11(22.0)	11(18.6)	3(6.3)	6(12.5)	0(0.0)	31(11.9)	4(8.5)	0(0.0)	2(3.9)	0(0.0)	0(0.0)	6(2.6)	37(7.5)
	Sometimes	38(76.0)	45(76.3)	44(91.7)	37(77.1)	48(88.9)	212(81.9)	23(48.9)	51(100.0)	24(47.1)	49(100.0)	33(97.1)	180(77.6)	392(79.8)
Wash hand after defecation n (%)	Always	7(14.0)	4(6.8)	3(6.3)	1(6.3)	0(0.0)	15(5.8)	6(12.8)	0(0.0)	0(0.0)	1(2.0)	0(0.0)	7(3.0)	20(4.9)
	Never	1(2.0)	1(1.7)	1(2.1)	2(4.2)	1(1.9)	6(2.31)	17(36.2)	0(0.0)	27(52.9)	0(0.0)	0(0.0)	44(18.9)	76(10.2)
	Sometimes	42(84.0)	54(91.5)	44(91.7)	43(89.6)	53(98.1)	238(91.9)	24(51.1)	51(100)	24(47.1)	48(98.0)	34(100)	53(78.0)	395(84.9)
Wash fruits before eating n (%)	Always	6(12.0)	5(8.5)	3(6.3)	1(2.1)	0(0.0)	15(5.8)	5(10.6)	0(0.0)	0(0.00)	0(0.0)	0(0.0)	5(2.2)	20(4.1)
	Never	3(6.0)	8(13.5)	8(16.7)	5(10.4)	4(7.4)	28(10.8)	18(38.3)	0(0.0)	27(52.9)	3(6.1)	0(0.0)	48(20.7)	76(15.5)
	Sometimes	41(82.0)	46(78.0)	37(77.1)	42(87.5)	50(92.6)	216(83.4)	24(51.1)	51(100)	24(47.1)	46(93.3)	34(100)	179(77.1)	395(80.4)

School 1 = Catholic school Mere-Regina, School 2 = Primary school Adjela, School 3 = Primary school Ekoum, School 4 = Primary bilingual school of Zoulabot, School 5 = Primary school Mintoum, School 6 = Primary school Manyang, School 7 = Primary school Mbalam, School 8 = Primary school Bosquet, School 9 = Primary school Messock, School 10 = Primary school Bareko,

### Therapeutic coverage in Lomie district

A total of 96.1% (95% CI: 94.1–97.6%) of children reported to have swallowed mebendazole at least once in the year preceding the survey (2022), with no significant difference between the sexes and schools for compliance with the drug. This proportion of children who experienced mebendazole uptake corresponded to the reports from the school registers (registered coverages ranging from 96 to 100%) and is not significantly different with the ESPEN report on the mean of the therapeutic coverage in this district the same year prior to the survey (Fig 2). On the other hand, leftover medication was found in 04 schools and all the school headmasters reported numerous cases of multiple treatments beyond the deworming campaign, particularly for children who had symptoms of STH infection at school (mainly abdominal pains). However, this therapeutic coverage was not uniform across the district, as the proportion of children who reported have taken the mebendazole the previous year is increased in CDTI areas (98.7%) compared to non-CDTI areas (93.8%) (Chi-squared = 6.6, df = 1, *p-value* = 0.01). With regard to ivermectin uptake in the CDTI areas, 64.6% (95% CI: 58.1–70.8%) of school-age children reported to have swallowed ivermectin the year preceding the survey, and the therapeutic coverage at the level community reached the 97% according to the ESPEN report (Fig 2).

### Prevalence of the STH infections

The overall prevalence of STHs among school-aged children was 83.9% (95% CI: 80.3%–87.1%) (Table 2). *T*. *trichiura* was the most prevalent STH infection and no hookworm species were reported. The overall prevalence of *T*. *trichiura* infection was 78.5% (95% CI: 74.5–82.1%) and that of *A*. *lumbricoides* infection was 62.7% (95% CI: 58.2–67.1%). Out of 405 STH infected children, 31.6% (95% CI = 27.1–36.4%) had a single helminth infection (mono-parasitism), either *T*. *trichiura* (25.2%) or *A*. *lumbricoides* (6.4%) only. The prevalence of poly-parasitism meaning the proportion of *A*. *lumbricoides* and *T*. *trichiura* coinfected people was 57.3%. The prevalence of the STH infection was significantly school-dependent, varying from 100% to 44.0% (Chi-squared = 143.6, df = 9, *p-value* < 0.0001). When considering the CDTI status of health areas and health areas themselves, the prevalence of the STH infection was significantly higher in CDTI areas (96.5%) compared to non-CDTI areas (72.2%) (Chi-squared = 50.8, df = 1, *p-value* < 0.0001) and Messock was the most prevalent health area of the district with 97.7% of individuals infected amongst the school-age participants (Chi-squared = 53.3, df = 3, *p-value* < 0.0001).

**Table 2:** Prevalence of the STH infections in the surveyed schools of the Lomie Health District.

CDTI Status	Health areas	Schools	Total examined	Prevalence of STH infection n (%)				
				STH infection	Poly-parasitism	Mono-parasitism	Ascaris lumbricoides	Trichuris trichiura
**Non–CDTI health areas**	**Lomie**	School 1	50	22 (44.0)	07 (14.0)	15 (30.0)	09 (18.0)	20 (40.0)
		School 2	59	41 (74.5)	20 (36.4)	21 (38.2)	25 (45.5)	36 (65.5)
		School 3	48	45 (95.7)	26 (55.3)	19 (40.4)	29 (61.7)	42 (89.4)
		Total Lomie	147	108 (71.1)	53 (34.9)	55 (36.2)	63 (41.7)	98 (64.5)
	**Zoulabot 1**	School 4	48	23 (48.9)	10 (21.3)	13 (27.7)	13 (27.7)	20 (42.6)
		School 5	54	51 (96.2)	38 (71.7)	13 (24.5)	43 (81.1)	46 (86.8)
		Total Zoulabot	102	74 (74.0)	48 (48.0)	26 (26.0)	56 (56.0)	66 (66.0)
	**Total Non CDTI areas**		**242**	**182 (72.2)**	**101 (40.1)**	**81 (32.1)**	**119 (47.2)**	**164 (65.1)**
**CDTI health areas**	**Ngoyla**	School 6	47	44 (93.6)	34 (72.3)	10 (21.3)	35 (74.5)	43 (91.5)
		School 7	51	49 (96.1)	29 (56.9)	20 (39.2)	33 (64.7)	45 (88.2)
		Total Ngoyla	98	93 (94.9)	63 (64.3)	30 (30.6)	68 (69.4)	88 (89.8)
	**Messock**	School 8	51	51 (100)	47 (92.2)	04 (7.8)	47 (92.2)	51(100)
		School 9	49	47 (95.9)	40 (81.6)	07 (14.3)	42 (85.7)	45 (91.8)
		School 10	34	32 (97.0)	26 (78.8)	06 (18.2)	27 (81.8)	31 (93.9)
		Total Messock	134	130 (97.7)	113 (85.0)	17 (12.8)	116 (87.2)	127 (95.5)
	**Total CDTI areas**		**232**	**223 (96.5)**	**176 (76.2)**	**47 (20.3)**	**184 (79.7)**	**215 (93.1)**
		**Overall**	**491**	**405 (83.9)**	**277 (57.3)**	**128 (26.5)**	**303 (62.7)**	**379 (78.5)**

School 1 = Catholic school Mere-Regina, School 2 = Primary school Adjela, School 3 = Primary school Ekoum, School 4 = Primary bilingual school of Zoulabot, School 5 = Primary school Mintoum, School 6 = Primary school Manyang, School 7 = Primary school Mbalam, School 8 = Primary school Bosquet, School 9 = Primary school Messock, School 10 = Primary school Bareko,

### Intensity of the STH infection

**Fig 3:**
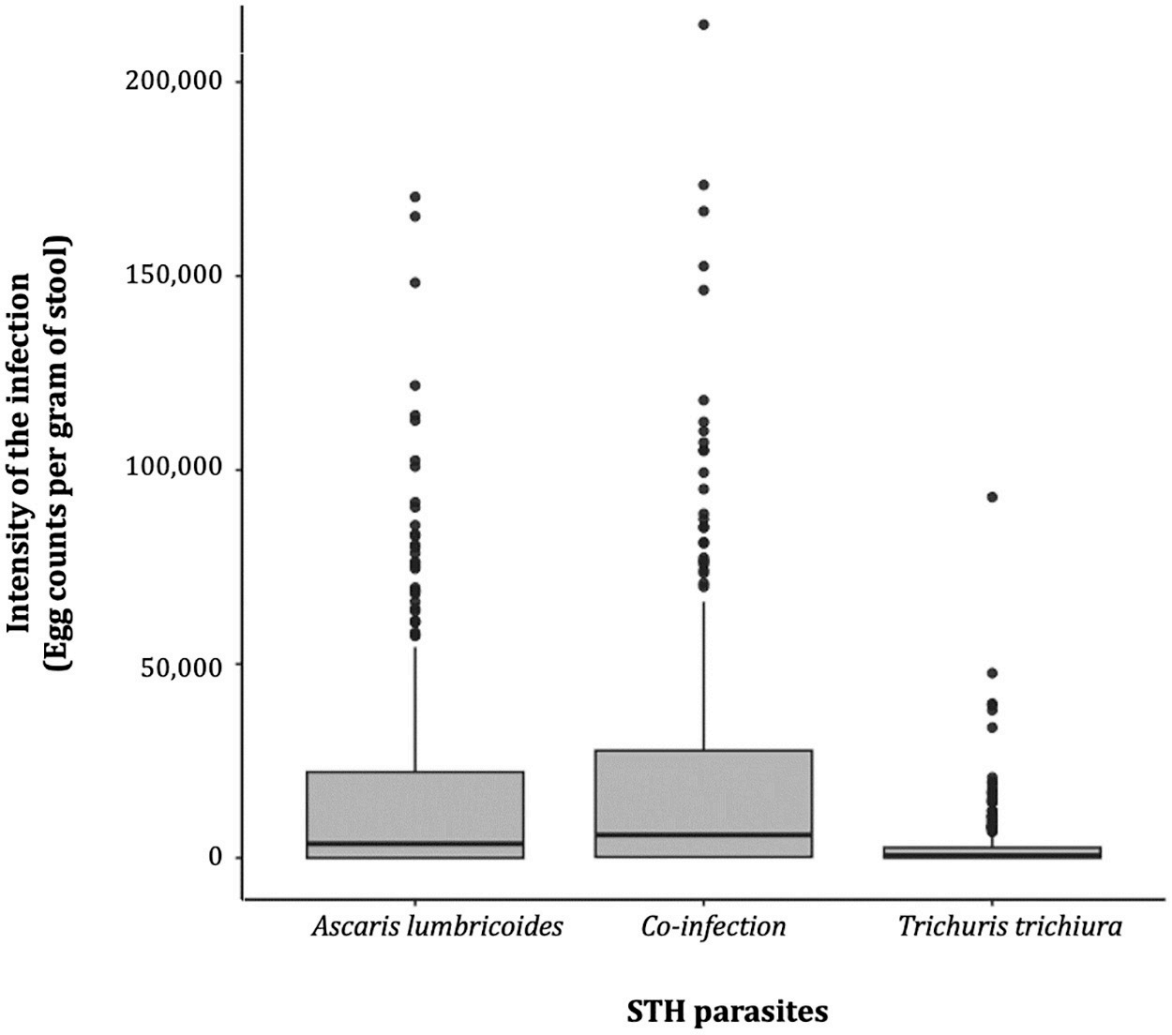
Distribution of parasite intensities of STH species and polyparasitism in children in the Lomie district.

The Fig 3 shows the distribution of the STH species and coinfection intensities amongst children in Lomie district. The arithmetic mean of egg per gram (EPG) of stool among STH-infected participants was 18101.02±27831.8 (ranging from 0 to 214824 EPG of stool). Considering the parasite’s species, the means of faecal egg count among infected children was 15354±24747.0 EPG (ranging from 0 to 170400 EPG of stool) and 2746.04 ±6675.2 EPG (intensity ranging from 0 to 93024 EPG of stool) for *A*. *lumbricoides* and *T*. *trichiura* respectively. The intensities of each parasite vary with regard to different variables. *A lumbricoides* were significantly higher in public schools (chi-squared = 77.6, df = 1, *p-value* < 0.0001) as well as the intensity of *T*. *trichiura* male (Chi-squared = 11.6, df = 1, *P-value* = 0.0006). The mean intensities of STH infection were significantly higher in CDTI areas compared to non-CDTI areas both for *A*. *lumbricoides* (Chi-squared = 61.1, df = 1, *p-value* < 0.0001) and *T*. *trichiura* (Chi-squared = 81.9, df = 1, *p-value* < 0.0001). Estimation of STH infection intensities based on the WHO classification of the egg count for each parasite showed that 7.7% of individuals were heavily infected with *A*. *lumbricoides* with the mean of faecal eggs count of 82,549.6 EPG of stool and 6.4% were heavily infected with *T*. *trichiura* with the mean of faecal eggs count of 20,904.0 EPG of stool (Table 3). The prevalence of heavily infected individuals was significantly higher in CDTI health areas for both *T*. *trichiura* (Chi-squared = 9.09, df = 1, *p-value* = 0.002) and A. lumbricoides infections (Chi-squared = 4.4, df = 1, *p*-value = 0.03), with estimated means of faecal eggs count of 91,279.2 EPG of stool (*A*. *lumbricoides*) and 16,149.6 EPG of stool (*T*. *trichiura*). As with the CDTI-based clusters, disaggregated data showed the heterogeneity of the distribution of heavy infections across the health areas and schools surveyed. Indeed, Ngoyla and Messock were the most prevalent health areas for individuals heavily infected with *A*. *lumbricoides* (prevalence:17.3%, mean intensity 86,538.3 EPG of stool) and *T*. *trichiura* (prevalence: 9.8%, mean intensity 18,422.7 EPG of stool) respectively, while the Lomie health area was the least prevalent. At school level, schools 6 (Ngoyla health area) and 8 (Messock health area), with 15.7% and 17.0% of children heavily infected with *A*. *lumbricoides* and *T*. *trichiura* respectively, represented the schools with the highest proportion of heavily infected children in the district for each parasite.

**Table 3:** Intensity of STH infections in Lomie Health District according to WHO classification.

CDTI Health areas	Schools	Intensity of the STH infection								
		Parameters	*A*. *lumbricoides*			Heavy	*T*. *trichiura*			Heavy
			Negative	Light	Moderate		Negative	Light	Moderate	
**Non-CDTI health areas**										
**Lomie**	School 1	n (%)	41 (82.0)	4 (8.0)	5 (10.0)	0 (0.0)	30 (0.0)	17 (34.0)	3 (6.0)	0 (0.0)
		Mean±SD	0.0 ±0.0	252.0±440.1	17318.4±11736.7	-	0.0 ±0.0	283.7±270.8	2760.0±2552.0	-
	School 2	n (%)	30 (54.5)	13 (23.6)	12 (21.8)	0 (0.0)	19 (34.5)	25 (45.0)	11 (20.0)	0 (0.0)
		Mean±SD	0.0 ±0.0	1755.6±1124.4	19250.0±1124.4	-	0.0 ±0.0	233.2±228.3	2727.2±1957.9	-
	School 3	n (%)	18 (38.3)	11 (23.4)	14 (29.8)	4 (2.1)	5 (10.6)	22 (46.8)	18 (38.3)	2 (4.3)
		Mean±SD	0.0 ±0.0	2064.0±1558.1	25695.4±12991.6	110970.0±56604.3	0.0 ±0.0	249.8±201.7	3974.6±2572.5	11412.0±933.3
	*Total Lomie*	***n (%)***	*89 (58*.*6)*	*28 (18*.*4)*	*31 (2*.*4)*	*4 (2*.*6)*	*54 (35*.*5%)*	*64 (42*.*1)*	*32 (21*.*1)*	*2 (1*.*3)*
		*Mean±SD*	*0*.*0 ±0*.*0*	*1662*.*0±1359*.*1*	*21849*.*3±13006*.*7*	*110970*.*0±56604*.*3*	*0*.*0 ±0*.*0*	*252*.*3±229*.*0*	*3432*.*0±2382*.*6*	*11412*.*0±933*.*3*
**Zoulabot**	School 4	n (%)	34 (72.3)	6 (12.8)	6 (12.8)	1 (2.1)	27 (57.4)	12 (25.5)	5 (10.6)	3 (6.3)
		Mean±SD	0.0 ±0.0	848±698.5	19600±12155.9	76392±NA	0.0 ±0.0	314±251.6	2520±1566.9	13008±2375.3
	School 5	n (%)	10 (18.9)	7 (13.2)	31 (58.5)	5 (9.4)	7 (13.2)	21 (39.6)	20 (37.7)	5 (9.4)
		Mean±SD	0.0 ±0.0	2643.4±1192.7	22397.4±13484.4	78504.0±15947.7	0.0 ±0.0	406.8±311.6	3175.2±1930.2	19929.6±15913.2
	*Sub-otal Zoulabot*	*n (%)*	*44 (44*.*0)*	*13 (15*.*9)*	*37 (39*.*1)*	*6 (7*.*7)*	*34 (34*.*0)*	*33 (33*.*0)*	*24 (25*.*0)*	*8 (8*.*0)*
		*Mean±SD*	*0*.*0 ±0*.*0*	*1814*.*7±1335*.*0*	*21943*.*7±13158*.*3*	*78152*.*0±14290*.*1*	*0*.*0 ±0*.*0*	*373*.*0±290*.*7*	*3044*.*1±1852*.*1*	*17334*.*0±12615*.*3*
***Total Non CDTI areas***		**n (%)**	**47 (20.3)**	**36 (15.9)**	**121 (52.4)**	**27 (11.7)**	**16 (6.9)**	**80 (34.6)**	**114 (49.4)**	**21 (9.1)**
		**Mean±SD**	**0.0±0.0**	**2517.3±1588.8**	**22414.2±12916.9)**	**79316.4±25886.9**	**0.0 ±0.0**	**286.4 ±281.6**	**3750.1± 2355.7**	**23168.0± 18619.5**
***CDTI health areas***										
**Ngoyla**	School 6	n (%)	12 (25.5)	7 (11.8)	20 (42.6)	8 (17.0)	4 (8.5)	24 (51.1)	18 (38.3)	1 (2.1)
		Mean±SD	0.0 ±0.0	2602.2±1762.6	23992.8±15310.9	73383.0±11226.8	0.0 ±0.0	485±254.5	2404±1045.5	19656±NA
	School 7	n (%)	18 (7.8)	6 (11.8)	18 (70.6)	17.6)	6 (11.8)	14 (27.5)	24 (47.5)	7 (13.7)
		Mean±SD	0.0 ±0.0	1620.0±1384.6	21454.6±15814.4	98232.0±33976.6	0.0 ±0.0	433.7±331.2	3982.0±2799.7	32482.2±28708.1
	*Total Ngoyla*	*n (%)*	*30 (30*.*6)*	*13 (13*.*3)*	*83 (38*.*8)*	*17 (17*.*3)*	*10 (10*.*2)*	*38 (38*.*8)*	*42 (42*.*9)*	*8 (8*.*0)*
		*Mean±SD*	*0*.*0 ±0*.*0*	*2148*.*9±1616*.*2*	*22790*.*5±15392*.*8*	*86538*.*3±28209*.*8*	*0*.*0 ±0*.*0*	*466*.*1±281*.*9*	*3305*.*7±2339*.*8*	*30879*.*0±26962*.*6*
**Messock**	School 8	n (%)	4 (7.8)	6 (22.4)	36 (59.1)	5 (9.8)	0 (0.0)	5 (9.8)	38 (74.5)	8 (15.7)
		Mean±SD	0.0 ±0.0	1804.0±1150.6	23574.6±10235.0	72624.0±18105.8	0.0 ±0.0	724.8±218.2	4830.3±2383.5	18393.0±9047.0
	School 9	n (%)	7 (14.3)	11 (15.9)	29 (39.1)	2 (7.7)	4 (8.2)	20 (36.6)	23 (35.4)	2 (6.4)
		Mean±SD	0.0 ±0.0	2762.1±1641.6	19204.9±11936.9	55992.0±6686.4	0.0 ±0.0	420.0±289.9	3259.8±2180.3	11472.0±882.4
	School 10	n (%)	6 (18.2)	6 (18.2)	18 (54.5)	3 (9.1)	2 (6.1)	17 (51.5)	11 (33.3)	3 (9.1)
		Mean±SD	0.0 ±0.0	3580.0±1495.2	24469.3±13655.9	65096.0±16268.9	0.0 ±0.0	392.4±284.5	2740.3±1357.3	23136.0±13027.2
	*Total Messock*	*n (%)*	*17 (12*.*8)*	*23 (17*.*3)*	*83 (62*.*4)*	*10 (7*.*5)*	*6 (4*.*5)*	*42 (31*.*6)*	*72 (54*.*1)*	*13 (9*.*8)*
		*Mean±SD*	*0*.*0 ±0*.*0*	*2725*.*5±1570*.*1*	*22241*.*9±11711*.*5*	*67039*.*2±15974*.*6*	*0*.*0 ±0*.*0*	*445*.*1±293*.*6*	*4009*.*3±2342*.*0*	*18422*.*7±9471*.*0*
***Total CDTI areas***		**n (%)**	**133 (52.8)**	**41 (16.3)**	**68 (27.0)**	**10(4.0)**	**88 (34.9)**	**97 (38.5)**	**57 (22.6)**	**10 (4.0)**
		**Mean±SD**	**0.0 ±0.0**	**1710.4 ±1505.7**	**21900.7 ±12991.7**	**91279.2 ±38323.3**	**0.0 ±0.0**	**293.4 ±256.7**	**3261.9± 2156.5**	**16149.6± 11406.7**
**Overall District**										
		**n (%)**	**180 (37.3)**	**77 (15.9)**	**189 (39.1)**	**37(7.7)**	**104 (21.5)**	**177 (36.6)**	**171 (35.4)**	**31 (6.4)**
		**Mean±SD**	**0.0 ±0.0**	**2087.7 ±1505.7**	**22229.5 ±12911.7**	**82549.6 ±29667.5**	**0.0 (0.0)**	**366.5 (±281.6)**	**3587.3± 2296.5**	**20904.0± 16771.4**

**n (%):** number of individuals and prevalence in percentage; **Mean±SD:** Arithmetic mean of eggs per gram of stool ± Standard deviation; School 1 = Catholic school Mere-Regina, **School 2** = Primary school Adjela, **School 3** = Primary school Ekoum, **School 4** = Primary bilingual school of Zoulabot, **School 5** = Primary school Mintoum, **School 6** = Primary school Manyang, **School 7** = Primary school Mbalam, **School 8** = Primary school Bosquet, **School 9** = Primary school Messock, **School 10** = Primary school Bareko,

### Trends in the prevalence of the STH in Lomie District overtime

**Fig 4:**
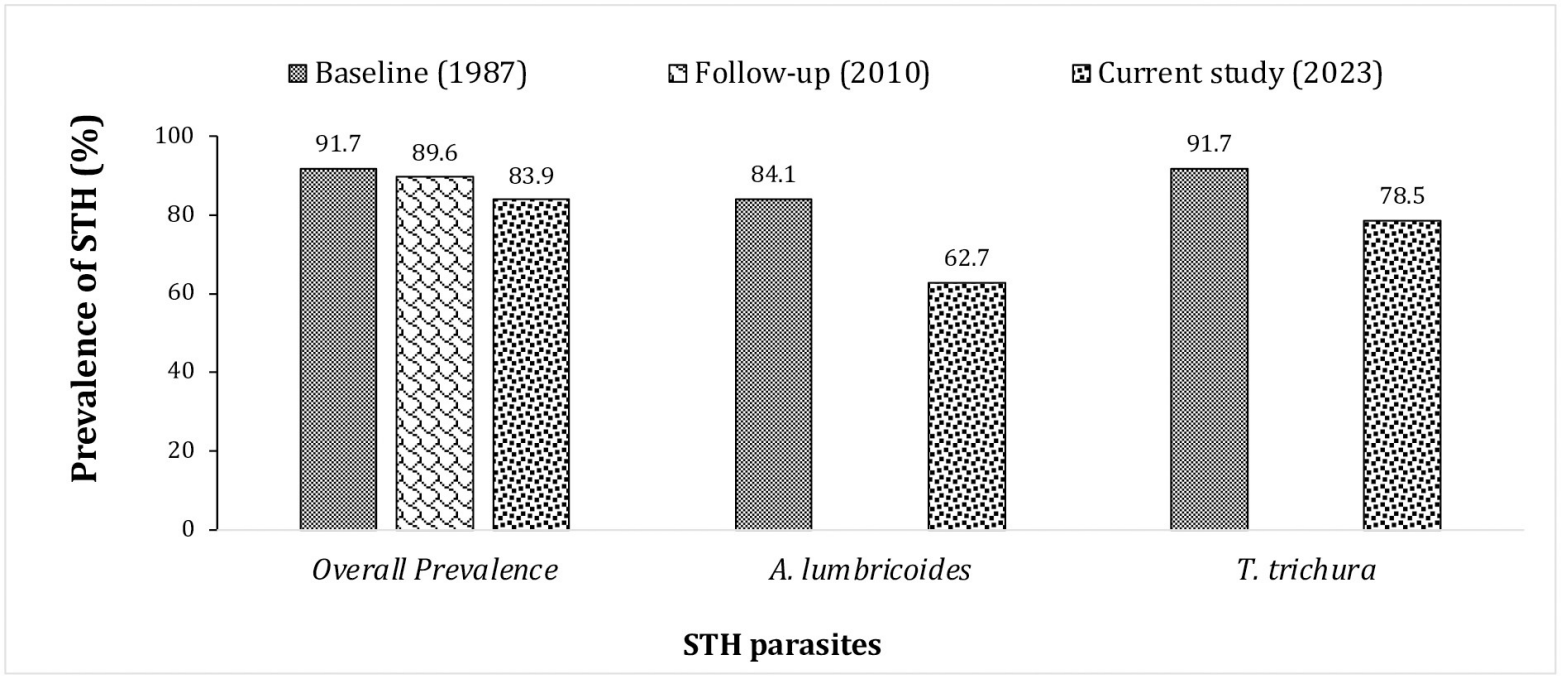
Comparison of STH infection rates prior the albendazole/mebendazole school-based treatments in 1987, considering the follow-up evaluation in 2010 (after 4 rounds of albendazole/mebendazole school-based treatments) and the current study (2023) in Lomie health district.

Fig 4 shows the comparison of the prevalence of STH infection determined in the current study, with the respective data from the 1987 epidemiological mapping conducted prior to the starting of the school-based preventive chemotherapy (baseline) [11,30], and the follow-up STH mapping conducted in 2010 by the control program (Schistosomiasis and STH National Control Programme). Less than 10% reduction in prevalence of STH was noted after almost 40 years since the baseline evaluation (prevalence dropped from 91.1% to 83.9% between 1987 and 2023). Considering the intervention era, 16 years of uninterrupted annual treatment of the school-age children (coupled to the community-based treatment of the other at-risk groups) with albendazole/mebendazole had no significant impact on the burden of STH in Lomie District (*p-value* = 0.05) (prevalence dropped from 89.6% to 83.9% between 2010 and 2023).

### STH infection with regard to at risk groups and at-risk behaviours

Table 4 shows the distribution of covariates between pupils with and without STH infection with regard to exposure to different risk factors. No association was found between the age group and the risk of being mono- or poly-infected, nor the high-moderate intensity of STH infection. However, males were significantly more infected than females, both in terms of mono- or polyparasitism and STH species. Hygiene practices also significantly influenced the prevalence of STH in the different groups. Poor hands and fruits washing habit, and always walking barefoot, significantly increased the prevalence of mono and polyparasitism and the infection by the parasite species *A*. *lumbricoides* and *T*. *trichiura* (*p-value* < 0.001). The source of the drinking water also affected the prevalence in the study population. People consuming water from rivers and wells were significantly more infected than those consuming water from other sources, both for the categories of infection (mono and polyparasitism, *p-value* < 0.0001) and for the STH species found (*A*. *lumbricoides* and *T*. *trichiura*). The distribution of the prevalence of moderate or high intensities of *A*. *lumbricoides* and *T*. *trichiura* species showed that children with dirty fingers (*p-value* = 0.0001), those who bite their nails (*p-value* = 0.0001), those who never washed their hands after defecation (*p-value* = 0.0001), those who walked barefoot and those who drink river water (*p-value* = 0.0001) had the highest prevalence of moderate or high intensity of STH among the surveyed population. Multivariate logistic regression (Table 5) showed that living in CDTI health areas of Lomie District, increased by 3.701 (95% IC: 1.3–12.3, *p-value* = 0.002) the risk of being infected by an STH parasite compared to the non-CDTI health areas. At the health areas level, with reference to Lomie, belonging to any other health area increased the risk of being infected by *A*. *lumbricoides* by 1.8 (in Zoulabot) to 9.6 (in Messock) times and by *T*. *trichiura* by 1.1 (in Ngoyla) to 11.7 (in Messock) times. The multivariate analysis also confirmed that having a river as a source of drinking water, increased to 14.8 (95% IC: 6.9–33.3, *p-value* = 0.0001) and 3.57 (95 IC: 1.0–11.3, *p-value* = 0.03) times the risk to be infected with STH than using pipe or well water. In terms of infection by parasite species, the risk of being infected by *A*. *lumbricoides* was 2.24 (95% IC: 1.4–3.4, *p-value* = 0.0002) times greater in children with dirty hands than in those with clean hands, 4.01 (95% IC: 1.3–12.2, *p-value* = 0.01)times greater in those who declared never washed their hands, and 3.65 (95 IC: 1.4–3.4, *p-value* = 0.005) times greater in those who didn’t systematically wash their hands after defecation than in those who reported always washing their hands. The same trend was observed for *T*. *trichiura* infections, where the risk of infection was significantly associated to male (OR: 1.98 [1.2–3.3], *p-value* = 0.009).

**Table 4:** Factors associated with soil transmitted helminths infections among school children in Lomie Health District.

Variable	Categories	Prevalence n (%)				Moderate-heavy STH intensity n (%)	
		STH infection	*A*. *lumbricoides*	*T*. *trichiura*	Polyparasitism	*A*. *lumbricoides*	*T*. *trichiura*
Age groups	5–9	242(81.5)	176(59.3)	226(76.1)	160(53.9)	133(44.8)	122(41.1)
	10–16	163(87.6)	127(68.3)	153(78.5)	117(62.9)	93(50.0)	80(43.0)
	**X**^**2**^ **(p)**	**2.74(0.09)**	**3.60(0.05)**	**2.2(0.13)**	**3.45(0.06)**	**1.05(0.30)**	**0.10(0.74)**
Sex	Females	182(79.8)	134(58.8)	164(71.9)	116(50.9)	101(44.3)	83(36.4)
	Males	233(87.5)	169(66.3)	215(84.3)	161(63.1)	125(49.0)	119(41.8)
	**X**^**2**^ **(p)**	**4.62(0.03)** *	**2.58(0.107)**	**10.2(0.001)** *	**6.904(0.008)** *	**0.89(0.34)**	**4.79(0.02)** *
Fingernails cleanliness	Clean fingernails	150(77.9)	102(50.0)	148(72.5)	91(44.6)	74(36.3)	66(32.4)
	Dirty fingernails	246(88.2)	201(72.0)	231(82.8)	186(66.7)	152(54.5)	136(48.7)
	**X**^2^ (p)	**8.36(0.003)** *	**2.58(0.000)** **	**6.72(0.009)** *	**22.5(0.000)** **	**14.9(0.0001)** **	**(0.0004)** *
Fingernails bites	No	192(83.1)	135(58.4)	180(77.9)	123(53.2)	94(40.7)	88(38.1)
	Yes	213(84.5)	168(66.7)	199(78.5)	154(61.1)	132(52.4)	114(45.2)
	**X**^**2**^ **(p)**	**0.08(0.76)**	**3.14(0.07)**	**0.02(0.86)**	**2.73(0.09)**	**6.1(0.01)** *	**2.24(0.13)**
Wash hand after defecation	Always	15(65.2)	10(43.5)	15(65.2)	10(43.5)	7(30.4)	7(30.4)
	Never	49(98.0)	42(84.0)	48(96.0)	41(82.0)	37(74.0)	35(70.0)
	Sometimes	341(83.0)	251(61.2)	316(78.5)	226(55.1)	182(46.8)	160(39.0)
	**X**^**2**^ **(p)**	**13.4(0.001)** *	**13.7(0.001)** *	**11.9(0.002)** *	**15.6(0.0005)** *	**18.3(0.0001)** *	**18.8(0.000)** **
Wash fruits and vegetable	Always	11(57.9)	8(42.1)	10(52.6)	7(36.8)	4(21.1)	5(26.3)
	Never	70(93.3)	57(76.0)	66(88.0)	53(70.7)	46(61.3)	42(56.0)
	Sometimes	324(83.9)	238(62.7)	303(77.9)	217(55.8)	176(45.2)	155(39.8)
	**X**^**2**^ **(p)**	**14.5(0.0007)** *	**(0.008)** *	**(0.003)** *	**9.09(0.01)** *	**11.8(0.002)** *	**8.6(0.01)** *
Walk barefoot	Always	5(91.9)	50(80.6)	56(90.3)	49(79.0)	43(69.4)	37(59.7)
	Never	11(69.4)	14(38.9)	24(66.7)	13(36.1)	8(22.2)	9(25.0)
	Sometimes	62(83.9)	239(62.1)	299(77.7)	215(55.8)	175(45.5)	156(40.5)
	**X**^**2**^ **(p)**	**8.5(0.01)** *	**17.3(0.0001)** *	**8.2(0.01)** *	**18.9(0.000)** **	**21.6(0.000)** **	**12.5(0.001)** *
Water source	Borehole	134(88.2)	97(63.8)	123(80.9)	86(56.6)	68(44.7)	55(36.2)
	Bottled	0(0.0)	0(0.0)	0(0.0)	0(0.0)	0(0.0)	0(0.0)
	Pipe	35(47.9)	16(21.9)	32(43.8)	13(17.8)	7(9.6)	11(15.1)
	Rain	1(50.0)	1(50.0)	1(50.0)	1(50.0)	1(50.0)	0(0.0)
	River	188(93.5)	146(72.6)	177(88.1)	135(67.2)	116(57.7)	103(51.2)
	Well	47(87.0)	43(79.6)	46(85.2)	12(77.8)	34(63.0)	33(61.1)
	**X**^**2**^ **(p)**	**0.000** **	**0.000** **	**0.000** **	**0.000** **	**0.000** **	**0.000** **

*Significant difference with P < 0.05;

**Significant difference with P < 0.01

**Table 5:** Multivariate Logistic regression analysis of STH infection amongst school children in Lomie Health District.

Variables	STH infection				*A*. *lumbricoides*				*T*. *trichiura*			
	OR [95% CI]	*p-value*	Adjusted-OR [95% CI]	*p-value* AOR	OR [95% CI]	*p-value*	Adjusted -OR [95% CI]	*p-value* AOR	OR [95% CI]	*p-value*	Adjusted -OR [95% CI]	*p-value* AOR
**Gender**												
*Male vs Female*	0.6 [0.3–0.9]	0.02*	0.62 [0.3–1.2]	0.19	1.37 [0.9–1.9]	0.08	1.13 [0.7–1.7]	0.58	2.09 [1.3–3.3]	0.001*	1.98 [1.2–3.3]	0.009*
**Age groups**												
*[5–9] vs [10 – 16]*	0.7 [0.3–1.03]	0.07	0.58 [0.3–1.2]	0.10	1.4 [0.9–1.9]	0.04*	1.63 [1.0–2.5]	0.03*	1.4 [0.9–2.3]	0.11	1.34 [0.8–2.3]	0.28
**Fingers cleanliness**												
*Clean vs dirty*	0.5 [0.3–0.8]	0.002*	0.95 [0.5–1.8]	0.88	2.6 [1.8–3.8]	0.000*	2.24 [1.4–3.4]	0.0002*	1.8 [1.2–2.8]	0.007*	1.12 [0.7–1.9]	0.66
**Fingernails bites**												
*No vs Yes*	0.9 [0.6–1.5]	0.67	1.1 [0.6–1.95]	0.84	1.4 [0.9–2.1]	0.06	1.29 [0.84–1.9]	0.23	1.1 [0.7–1.6]	0.78	0.88 [0.5–1.5]	0.62
Walk barefoot												
*Never vs always*	5.01 [1.6–17.3]	0.006*	1.13 [0.3–5.1]	0.86	0.1 [0.06–0.4]	0.000*	0.44 [0.1–1.2]	0.13	0.2 [0.0–0.6]	0.005*	0.93 [0.2–3.6]	0.91
*Sometimes vs always*	2.1 [0.9–6.5]	0.10	1.13 [0.34–4.3]	0.93	0.4 [0.2–0.7]	0.005*	0.60 [0.2–1.4]	0.26	0.4 [0.1–0.8]	0.02*	0.85 [0.3–2.5]	0.76
**Hand wash after toilet**												
*Never vs always*	0.01 [0.0–0.08]	0.0001*	0.01 [0.0–0.09]	0.0001*	7.06 [2.8–18.9]	0.000*	4.01 [1.3–12.2]	0.01*	76 [13.9–943]	0.000*	77 [12.4–153]	0.0001*
*Sometimes vs always*	0.19 [0.08–0.4]	0.0001*	0.18 [0.07–0.4]	0.0004*	3.3 [1.5–7.6]	0.002*	3.65 [1.4–9.4]	0.005*	3.9 [1.8–8.4]	0.0004*	3.7 [1.5–9.3]	0.004*
**Sources of water**												
*Borehole vs river*	1.9 [0.9–4.2]	0.08	1.91 [0.9–4.2]	0.31	0.6 [0.9–4.2]	0.07	0.70 [0.4–1.1]	0.14	0.5 [0.3–1.03]	0.06	0.54 [0.3–1.0]	0.04
*Bottled vs river*	3E7 [0.0 –NA]	0.98	1.7E7 [0.0 –NA]	0.99	4E-7 [NA– 1E9]	0.97	0.00 [NA]	0.97	1E-7 [NA– 4E]	0.97	0.00 [NA]	0.98
*Pipe vs river*	15 [7.7–33.5]	0.000*	7.8 [3.4–18.3]	0.000*	0.1 [0.05–0.2]	0.000*	0.14 [0.06–0.2]	0.000*	0.1 [0.05–0.2]	0.000*	0.10 [0.1–0.2]	0.000*
*Rain vs river*	14 [0.5–380]	0.06	5 [0.18–137]	0.28	0.4 [0.01–9.6]	0.49	0.30 [0.01–7.7]	0.40	0.1 [0.01–3.5]	0.16	0.21 [0.0–5.6]	0.28
*Well vs river*	2.15 [0.7–5.5]	0.12	2.14 [0.6–6.9]	0.21	1.5 [0.7–3.2]	0.3	1.37 [0.5–3.4]	0.47	0.8 [0.01–1.9]	0.57	0.35 [0.1–1.1]	0.05
***CDTI Status of the HA***												
*Non CDTI vs CDTI*	10.7 [5.3–24.7]	0.000*	3.7 [1.3–12.3]	0.02*	0.2 [0.1–0.3]	0.000*	0.5 [0.2–0.9]	0.02*	0.1 [0.07–0.2]	0.000*	0.31 [0.1–0.7]	0.006*
**Health Areas**												
*Messock vs Lomie*	0.05 [0.01–1.2]	0.000*	0.6 [1.2–2.9]	0.57	9.6 [0.5–0.9]	0.000*	2.5 [1.2–5.3]	0.01*	11.7 [5.2–1.9]	0.000*	1.74 [0.6–5.7]	0.33
*Ngoyla vs Lomie*	1.1 [0.04–0.3]	0.000*	0.00 [NA]	NA	3.2 [5.4–18.1]	0.000*	0.00 [NA]	NA	4.8 [2.4–10.6]	0.000*	0.00 [NA]	NA
*Zoulabot 1 vs Lomie*	0.9 [0.5–1.5]	0.60	0.8 [0.4–1.65]	0.8	1.8 [1.1–3.0]	0.02*	1.7 [0.9–3.1]	0.07	1.1 [0.6–1.8]	0.80	1.08 [0.57–2.1]	0.80

**AOR**: Adjusted Odd Ratio; **OR**: Odd-ratio

*Significant difference with P < 0.05

## Discussion

Soil transmitted helminth (STH) infections are among the most common NTDs, primarily affecting children living in the poorest settings with low access to WASH conditions in low-income countries [31]. The present study aimed to assess the epidemiological situation of the STH transmission in Lomie health district after long-term school-based annual deworming campaigns with mebendazole, in a context where community-directed treatment with ivermectin was also ongoing for the control of onchocerciasis.

The results revealed a very high prevalence of STH infections (83.9%). Although all children were mebendazole-based mass dewormed four months prior to the survey with 96.1% of therapeutic coverage, the disaggregated data showed that 70% of the schools presented a prevalence higher than 90%. This STH prevalence is the highest amongst the findings from all the epidemiological updates across the country [14,32–36]. This high prevalence shows the ineffectiveness of current mebendazole deworming campaigns in Lomie District even with satisfactory coverages rates. It reflects the high re-infection rates amongst children living in this district and their continue exposure to at-risk factors associated with STH transmission. Indeed, most students who do not have latrines at home or at school reported defecating in the bushes that usually surround the river or in the playgrounds, thereby disseminating STH eggs into the environment. In addition, analysis of behavioural determinants showed that factors significantly associated with infection included the use of the river as a source of drinking water, poor hand hygiene, and the habit of walking barefoot. These habits increase contact with contaminated soil when playing in outdoor environment, the risk of infection and the maintains of the parasite transmission cycle as reported in previous studies [5,6,37]. Furthermore, long-term exposure to anthelmintic drugs could be associated with the emergence of drug-resistant parasites in this area. This hypothesis justifies the need for further research to establish the polymorphism of beta-tubulin, a marker associated with resistance to anthelmintic drugs [38–40].

Comparison of the burden of STH between schools belonging to the health areas under ivermectin-based mass treatments (CDTI health areas) and those of the non-CDTI health areas, showed that the prevalence and intensities of STH (irrespective of parasite species) were significantly higher in CDTI health areas. This unexpected and counter-intuitive result contrasts with numerous comparative studies that have reported collateral impacts of ivermectin on STH during mass treatments against onchocerciasis or lymphatic filariasis [14,15,17,41,42]. This observation may be explained by differences in the intensity of infection and the behaviour of pupils in the two zones. Indeed, given the high intensity of infection among children living in CDTI health zones and their potential for parasite dissemination due to their high at-risk behaviours, the expected number of secondary cases produced by a single infection should be considerably higher than in non-CDTI health zones. These differences have led to a higher force of infection in CDTI health areas, which appears to offset the expected collateral impact of community treatment with ivermectin, revealing the existence of micro-contexts that should be considered to refine the understanding of disease dynamics within the health district.

This hypothesis is reinforced by disaggregated data at the level of health areas and schools, which indicate significant heterogeneity in the distribution of the disease, with the presence of hotspot health areas (Messock and Ngoyla) and hotspot schools (where all children are infected, including schools 6 and 8) which present a significant risk of infection and continued transmission of STH in the Lomie District. Further investigation should be carried out to determine whether the level of transmission in schools reflects the situation in the community and to identify potential at-risk groups, including out-of-school school-age children not targeted by the mebendazole MDA and who could act as reservoirs for sustained transmission.

The comparison of the current prevalence with data collected almost 40 years earlier (in 1987) [11] showed less than 8% decrease in STH prevalence in the district and, since the commencement of albendazole/mebendazole PCT, no significant change in prevalence is noted. The inter-sites comparison indicated that the impact of PCT on STH prevalence in Lomie is substantially lower than in other districts with similar baseline data and where the same interventions (at-risk groups or community-based treatments with mebendazole, albendazole and ivermectin) have been implemented. These include the health districts of Loum, Tiko and Yabassi, where the reduction thresholds vary between 80% and 95% over the same timeframe [11,14,34,35] However, the difference in the bio-ecological features of the health districts compared, partly explains the intensity of change in STH transmission, since the Ndelele Health District, which belongs to the same forest area with Equatoguinean climate as Lomie, showed a less pronounced reduction in transmission during the years of treatment [43]. Therefore, in addition to the history of preventive chemotherapy, potential collateral impacts of other health interventions and behavioural and structural determinants, this study highlights the importance of the bio-ecological features of the study area as context-dependent factors involved in the maintenance of STH transmission. Beyond these hypotheses, it appears necessary for the control programme to be aligned with WHO recommendations, which state that, once the prevalence rate exceeds 50% in schools, treatment should be extended beyond school-age children and other at-risk groups to the whole community and reinforce the water sanitation and hygiene (WASH) [29].

*A*. *lumbricoides* and *T*. *trichiura* were the only STH species represented and no hookworm was identified. These STH species are known to be overrepresented in STH infections in tropical areas. [4]. This can also be explained by the basic reproductive number (Ro) which represents the average number of female offsprings produced by one adult female parasite that attain the maturity in absence of density dependent constraints [44,45]. The Ro value is intrinsically highest for *T*. *trichiura* and *A*. *lumbricoides* and lowest for hookworm species and can therefore explain the usual discrepancy of species distribution in epidemiological studies [46]. The other justification for the higher prevalence of *A*. *lumbricoides* and *T*. *trichiura* in the study area could possibly be the humid climatic condition and clay nature of the soil which is favourable for the ova to survive longer [47].

## Conclusion

The persistent high prevalence and intensity of STH infection despite 16 years of mebendazole-based PCT and expected beneficial impact of ivermectin mass distribution points to plausible implementation gaps, poor WASH structures and hygiene practices or efficacy of the anthelminthics used. This study highlights the need to further assess the cause of the persistent high prevalence and implement context-adapted control measures in order to curb STH transmission.

## Supporting information

S1 Text Semi-structured questionnaire. (DOCX)
