# Supplementary material for: Persistent transmission of soil-transmitted helminths despite 16 years of uninterrupted Mebendazole- and ivermectin-based preventive chemotherapy in the Lomie Health District (East Region, Cameroon): The emergency of complementary control strategies
Source: PLoS Negl Trop Dis. 2024 Sep 25;18(9):e0012508. doi: 10.1371/journal.pntd.0012508 (PMC11476675; doi:10.1371/journal.pntd.0012508)
Supplement: S1 Text — (DOCX) [file pntd.0012508.s001.docx]

# Questionnaire for school-based study

# Ivermectin-based community mass treatment: Perspective for accelerating soil-transmitted helminths elimination in Cameroon and Ghana

|  | **GENERAL INFORMATION** | |
| --- | --- | --- |
|  | Country | Ghana |
|  |  | Cameroon |
|  | Region | Volta |
|  |  | East Akim |
|  |  | Littoral |
|  |  | Eastern region |
|  | Health District | Keta |
|  |  | Abuakwa South |
|  |  | Ndom |
|  |  | Lomie |
|  | GPS Coordinates | Lat:………………………. |
|  |  | Long:……………………. |
|  | Health Area: |  |
|  | School Name |  |
|  | School ID (Generated by software) |  |
|  | Community/Village school is located |  |
|  | Village ID(Generated by software) |  |
|  | N° rank: |  |
|  | Final ID (School ID + N° rank): |  |
|  | Participant Unique ID (Generated by software) |  |
|  | Interviewer Name |  |
|  | Date of interview | …… …… …………  dd mm year |
|  | **HAS SAMPLE BEEN COLLECTED?** | **YES** |
|  |  | **NO** |
|  | **School characteristics (Interviewer to observe and document)** | |
|  | Is there a toilet facility in the school for children | Yes |
|  |  | No |
|  | If yes, specify type of toilet | Water closet (Flush toilet) |
|  |  | KVIP |
|  | Is there a handwashing facility for the toilet? | Yes |
|  |  | No |
|  | **Socio-Demographic Characteristics** | |
|  | Sex | Male |
|  |  | Female |
|  | Age at last birthday (Years) |  |
|  | Class or form | Two (2) |
|  |  | Three (3) |
|  |  | Four (4) |
|  |  | Five (5) |
|  |  | Six (6) |
|  | Religion | Christian |
|  |  | Muslim |
|  |  | Tradional |
|  |  | Other (Specify) |
|  | Who do you live with? | Mother only |
|  |  | Father only |
|  |  | Both Mother and Father |
|  |  | Sister only |
|  |  | Brother only |
|  |  | Grand parents |
|  |  | Other relatives |
|  | Occupation of mother  **NB: This question is applicable if the child lives both mother and father or just mother** | Trader |
|  |  | Farmer |
|  |  | Teacher |
|  |  | Nurse/health worker |
|  |  | Doctor |
|  |  | Unemployed |
|  |  | Don’t know |
|  | Occupation of father  **NB: This question is applicable if the child lives both mother and father or just father** | Trader |
|  |  | Farmer |
|  |  | Teacher |
|  |  | Nurse/health worker |
|  |  | Doctor |
|  |  | Unemployed |
|  |  | Don’t know |
|  | Occupation of guardian  NB: this question is only applicable if the child does not live with either mother or father | Trader |
|  |  | Farmer |
|  |  | Teacher |
|  |  | Nurse/health worker |
|  |  | Doctor |
|  |  | Unemployed |
|  |  | Don’t know |
|  |  |  |
|  | **Anthropometric measurement / ITN use/ Nail hygiene** | |
|  | Did you sleep under a mosquito net last night? | Yes |
|  |  | No |
|  | Weight (kg) | ………………………….. |
|  | Height (centimeters) | ………………………….. |
|  | Mid arm circumference (centimeters) | …………………………. |
|  | Observe childs hands to ascertain dirt in finger nails | Finger nails with dirt |
|  |  | Finger nails without dirt |
| **Attitudes and practices related to the prevention/control measures** | | |
|  | Do you usually wash your hands before eating? | Always |
|  |  | Sometimes |
|  |  | Never |
|  | Do you usually wash fruits (e.g mango, pawpaw) before eating it | Always |
|  |  | Sometimes |
|  |  | Never |
|  | Do you wash your hands after using the toilet? | Always |
|  |  | Sometimes |
|  |  | Never |
|  | If yes to 16, do you wash your hands with soap? | Always |
|  |  | Sometimes |
|  |  | Never |
|  | Do you usually walk barefoot? | Always |
|  |  | Sometimes |
|  |  | Never |
|  | Do you eat dirt (white clay, mud)? | Yes |
|  |  | No |
|  | Do you bite your nails? | Yes |
|  |  | No |
|  | What source of water do you drink at home? | Tap/pipe |
|  |  | Borehole |
|  |  | Well |
|  |  | Sachet water |
|  |  | Bottled |
|  |  | Rain |
|  |  | River or stream |
|  | Do you bath or swim in river or stream? | Yes |
|  |  | No |
|  | Is there a toilet facility in your household? | Yes |
|  |  | No |
|  | If yes, specify type | Water closet/flush |
|  |  | KVIP |
|  | If no, where do you ease yourself? | Nearby bush |
|  |  | Seaside |
|  |  | Public toilet |
|  | History of treatments (ivermectin, albendazole, mebendazole and praziquantel) | |
|  | Did you take albendazole last year? | Yes |
|  |  | No |
|  | Did you take mebendazole last year? | Yes |
|  |  | No |
|  | Did you take ivermectin last year? | Yes |
|  |  | No |
|  | Did you take praziquantel last year? | Yes |
|  |  | No |
|  | Number of treatments taken the last 5 years |  |
|  | albendazole | …………….. |
|  | mebendazole | ……………… |
|  | ivermectin | ………………. |
|  | praziquantel | ……………… |
|  |  |  |
|  | **Morbidity related to STH infections** | |
|  | Do you have diarrhoeas/ Liquid Stool? | Yes |
|  |  | No |
|  | Do you experience nausea/vomiting? | Yes |
|  |  | No |
|  | Do you experience stomach pains | Yes |
|  |  | No |
|  | Do you see blood in your stool? | Yes |
|  |  | No |
|  | Do you see mucus in your stool? | Yes |
|  |  | No |
|  | Do you have lack of appetite? | Yes |
|  |  | No |
|  | Do you experience an urgent need to have a bowel movement with little or no stool? | Yes |
|  |  | No |
|  | Do you experience anal pain when having a bowel movement? | Yes |
|  |  | No |
|  | Do you experience anal itching? | Yes |
|  |  | No |
|  | NOTES OR OTHER OBSERVATIONS IF ANY | |
|  | ……………………………………………………………………………… | |
